# Government Policies to Reduce Free or Added Sugars and Use Nonsugar Sweeteners Should Support Plant-Rich Dietary Patterns, a Healthy Microbiome and Planetary Health

**DOI:** 10.1016/j.cdnut.2024.103788

**Published:** 2024-05-25

**Authors:** Vivica I Kraak, Nicole A Leary

**Affiliations:** From the Department of Human Nutrition, Foods, and Exercise, Virginia Polytechnic Institute and State University (Virginia Tech), Blacksburg, VA, United States

Dear Editor:

Policy coherence reinforces actions across government departments and agencies to create synergies that achieve objectives to maximize opportunities, expose trade-offs, and minimize inconsistencies to support the collective impact of policies [1]. Policy coherence is important for national governments to ensure that businesses follow evidence-informed guidelines to reduce free or added sugars and whether to replace added or free sugars in products with artificial or natural nonsugar sweeteners (NSSs), in order to support minimally processed plant-rich dietary patterns, a healthy microbiome, and planetary health. Planetary health represents the health of humans and the natural systems (i.e., animals and ecosystems) on which they depend.

In 2024, *Current Developments in Nutrition* published 2 articles about added sugars and nonnutritive sweeteners. Coyle et al. [2] highlighted the importance of governments using a comprehensive definition for sugars linked to poor health to inform national food policy but did not address NSS policies. Chatelan et al. [3] explored whether governments should advise individuals to replace sugary beverages with low calorie–sweetened beverages containing nonnutritive sweeteners (hereafter, called NSS) to prevent obesity and cardiometabolic diseases but did not address sugary beverage policies [3]. *Current Developments in Nutrition* also has a special issue (https://cdn.nutrition.org/cdnut-enabling-transformation-of-food-systems) on food systems transformation to support the United Nations (UN) Sustainable Development Goals 2030 Agenda (SDG). Several UN agencies defined food systems transformation as the “need for change to be intentional and profound, based on factual understandings and societal agreements, and aimed at achieving outcomes at scale” [4].

NSS have become ubiquitous in thousands of packaged food and beverage products in the global food supply. The global NSS market is expected to reach $26 billion United States dollars by 2030 [5]. Russell et al. [6] analyzed the per capita volume of added sugars and NSS from beverage sales globally (2007–2019) and found a 36% per capita volume increase in global NSS sales, a reduction in added sugars from beverages in high-income countries, and an increase in added sugars in upper-income and lower middle-income countries.

These global trends suggest that national governments must prioritize policy coherence to develop guidelines for populations and businesses to reduce both free or added sugars aligned with recommended targets (<10% of total daily energy intake) and the types and amounts of NSS to support a healthy human microbiome, healthy weight, and ecosystems [3,[7], [8], [9], [10], [11], [12]].

Consensus statements and reports issued by the WHO, expert committees, and professional societies have either encouraged or discouraged governments to develop NSS policies to help people with obesity and type 2 diabetes reduce calorie intake, support glycemic control, and manage weight [2,[6], [7], [8], [9], [10], [11], [12]]. Chatelan et al. [3] concluded that NSS benefits are small, may produce altered gut microbiomes, but outweigh potential cardiometabolic health harms. Although improving glucose concentrations and cardiometabolic health are important, research must also evaluate effective policies to support the UN’s food systems transformation efforts.

Coyle et al. [2], Chatelan et al. [3], and Russell et al. [10] emphasized that decision makers must examine the policy context, risks, and unintended consequences of NSS and added sugars that hinder healthy dietary patterns. If governments will not enact effective legislation, the transnational agrifood industry will accelerate using NSS in highly processed products at the expense of people’s health [3,[5], [6], [7], [8], [9], [10]]. As the evidence for artificial and natural sweeteners evolves, governments must reevaluate and update coherent policies informed by new evidence [[7], [8], [9], [10], [11]] to minimize policy inconsistencies; transition to minimally processed plant-rich dietary patterns; and transform agrifood systems for healthy people, ecosystems and planetary health.

In 2023, the WHO’s Nutrition Guidance Expert Advisory Group (NUGAG) found insufficient evidence, based on a 2022 systematic review of 283 studies, for NSS benefits to outweigh potential harms [11]. The WHO NUGAG recommended in a provisional report that healthy adults should not use NSS for weight control or management owing to potential adverse effects of long-term use, such as increased noncommunicable diseases morbidity and mortality [12]. No explicit WHO recommendation was issued for adults with type 2 diabetes, which affects 537 million adults (aged 20–79 y) globally, and may increase to 643 million by 2030 [13]. The WHO Director for Nutrition advised people to reduce sweetness in their diet to improve health, use naturally occurring sugars in fruit, or consume unsweetened foods and beverages [12,14].

UN agencies advise national governments to adopt best practices that address free sugars and NSS concurrently and must develop strong policy coherence for this issue. A 2019 FAO and WHO report described 16 principles for sustainable diets that encouraged governments to promote water as the healthy default beverage, limit free sugars to <10% of total daily energy, but did not mention NSS [15].

A 2019 compromise among the FAO and WHO Codex Alimentarius members led to General Standards for Food Additives that allow sweeteners to be used to reduce the energy content or enhance flavors in certain categories [16]. Policy incoherence generates policy inertia, which is a formidable challenge to transform food systems to mitigate the Global Syndemic of undernutrition, obesity, and climate change [17]. Replacing added sugars with NSS may be a short-term solution for dietary transition but is incompatible with minimally processed plant-rich dietary patterns and regenerative agrifood systems.

Therefore, relevant UN agencies should update the Codex guidelines to support policy coherence and advise governments to develop added sugar and NSS policies to maximize opportunities and minimize inconsistencies that will support sustainable diets, a healthy human microbiome, and food systems transformation [[10], [11], [12], [13], [14], [15], [16], [17]]. Governments and other decision makers must consider different political economy contexts to develop, implement, monitor, and evaluate the impact of coherent synergistic policies to transform diets and food systems for future generations and planetary health [7,10,17,18]. Such policies include the following: healthy beverage recommendations in national food-based dietary guidelines; sugary beverage taxes earmarked for health-promotion programs; safe, accessible, and free water; front-of-package labeling and warning labels to discourage sugary beverages; and media campaigns that encourage water consumption.

## Authors contributions

The authors’ responsibilities were as follows – VIK, NAL: conceptualized, researched, and coauthored the letter; VIK: submitted this letter; and both authors read and approved the final manuscript.

The authors report no conflicts of interest.
